# Ethyl­enediaminium hemioxalate thio­cyanate

**DOI:** 10.1107/S1600536810005994

**Published:** 2010-02-20

**Authors:** Leila Narimani, Bohari M. Yamin

**Affiliations:** a School of Chemical Sciences and Food Technology, Universiti Kebangsaan Malaysia, 43600 Bangi, Selangor, Malaysia

## Abstract

In the title compound, C_2_H_10_N_2_^2+^·0.5(C_2_O_4_)^2−^·NCS^−^, the ethyl­enediaminium dication adopts a (+)-synclinal conformation with an N—C—C—N torsion angle of 62.64 (15)°. The oxalate dianion lies across an inversion centre. In the crystal structure, the ions are linked through N—H⋯N, N—H⋯O and C—H⋯S hydrogen bonds, leading to the formation of a three-dimensional network.

## Related literature

For related structures, see: Barnes *et al.* (1998 ▶); Smith *et al.* (2006 ▶); Seidel *et al.* (2008 ▶); Tang *et al.* (2009 ▶). For bond-length data, see: Allen *et al.* (1987 ▶).
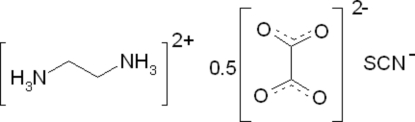


## Experimental

### 

#### Crystal data

C_2_H_10_N_2_^2+^·0.5C_2_O_4_^2−^·NCS^−^
*M* *_r_* = 164.21
Triclinic, 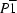

*a* = 6.4044 (19) Å
*b* = 6.6199 (19) Å
*c* = 9.377 (3) Å
α = 80.799 (5)°
β = 81.179 (5)°
γ = 74.452 (5)°
*V* = 375.5 (2) Å^3^
*Z* = 2
Mo *K*α radiation
μ = 0.38 mm^−1^
*T* = 298 K
0.43 × 0.41 × 0.35 mm

#### Data collection

Bruker SMART APEX CCD area-detector diffractometer
Absorption correction: multi-scan (*SADABS*; Sheldrick, 1996 ▶) *T*_min_ = 0.854, *T*_max_ = 0.879
5091 measured reflections
1867 independent reflections
1718 reflections with *I* > 2σ(*I*)
*R*_int_ = 0.017

#### Refinement

*R*[*F* ^2^ > 2σ(*F* ^2^)] = 0.034
*wR*(*F* ^2^) = 0.095
*S* = 1.06
1867 reflections
94 parameters
H-atom parameters constrained
Δρ_max_ = 0.51 e Å^−3^
Δρ_min_ = −0.52 e Å^−3^

### 

Data collection: *SMART* (Siemens, 1996 ▶); cell refinement: *SAINT* (Siemens, 1996 ▶); data reduction: *SAINT*; program(s) used to solve structure: *SHELXTL* (Sheldrick, 2008 ▶); program(s) used to refine structure: *SHELXTL*; molecular graphics: *SHELXTL* software used to prepare material for publication: *SHELXTL*, *PARST* (Nardelli, 1995 ▶) and *PLATON* (Spek, 2009 ▶).

## Supplementary Material

Crystal structure: contains datablocks global, I. DOI: 10.1107/S1600536810005994/ci5031sup1.cif

Structure factors: contains datablocks I. DOI: 10.1107/S1600536810005994/ci5031Isup2.hkl

Additional supplementary materials:  crystallographic information; 3D view; checkCIF report

## Figures and Tables

**Table 1:** Hydrogen-bond geometry (Å, °)

*D*—H⋯*A*	*D*—H	H⋯*A*	*D*⋯*A*	*D*—H⋯*A*
N2—H2*B*⋯N1	0.89	2.11	2.986 (2)	169
N3—H3*A*⋯O2	0.89	2.02	2.8685 (17)	158
C3—H3*E*⋯S1	0.97	2.77	3.7294 (18)	169
N2—H2*A*⋯N1^i^	0.89	2.05	2.899 (2)	159
N2—H2*C*⋯O1^ii^	0.89	1.95	2.8337 (18)	172
N3—H3*B*⋯O1^iii^	0.89	2.02	2.9078 (17)	174
N3—H3*C*⋯O1^ii^	0.89	2.40	3.0465 (18)	129
N3—H3*C*⋯O2^iv^	0.89	1.99	2.8189 (18)	154
C4—H4*B*⋯S1^v^	0.97	2.68	3.4495 (18)	136

Symmetry codes: (i) 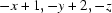; (ii) 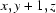; (iii) 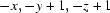; (iv) 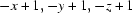; (v) 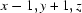.

**Fig. 1.**
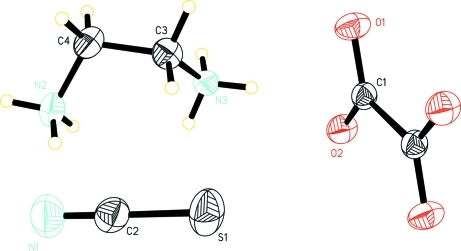
The asymmetric unit of the title compound. Displacement ellipsoids are drawn at the 50% probability level. Unlabelled atoms in the dianion are related to other labelled atoms in it by the symmetry operation (1 - x, -y, 1 - z).

**Fig. 2.**
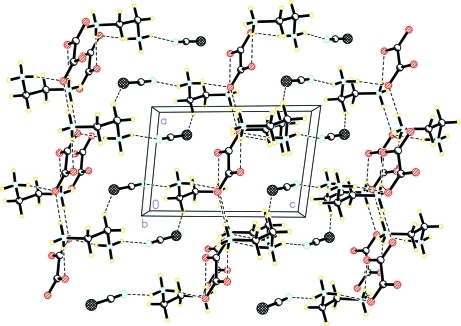
Packing diagram of the title compound, viewed down the b axis. Hydrogen bonds are shown as dashed lines.

## supplementary crystallographic information

### Comment

Aqueous solution of ethylenediamine and oxalic acid regardless of their stiochiometric ratio was reported to give ethylenediammonium bis(monohydrogen oxalate) monohydrate (II) (Barnes *et al.*, 1998). However, the same reaction but in the presence of ammonium thiocyanate was found to give an ethylenediammonium hemioxalate thiocynate, the title compound, (I, Fig.1), indicating that the oxalic acid has been completely deprotonated.

The centrosymmetric oxalate anion is planar as commonly observed in many oxalate salts (Tang *et al.*, 2009; Seidel *et al.*, 2008). The C—O bond lengths are quite similar indicating a delocalisation of electron about the O—C—O bond as observed in (II) and *N*-[2-(2-chlorophenyl)-2-hydroxyethyl]-propann-2-aminium hemioxalate (III) (Tang *et al.*, 2009). The ethylenediaminium ion in this salt is not planar but twisted with a N3—C3—C4—N2 torsion angle of 62.64 (15)°. In compound (II), and ethylenediammonium pyridine-2,5-dicarboxylate dihydrate (IV) (Smith *et al.*, 2006), the ethylenediammonium cation is centrosymmetric and has an extended conformation with a N—C—C—N torsion angle of 180°. The thiocyanate anion is linear. The bond lengths and angles are in normal ranges (Allen *et al.*, 1987) and comparable with those in (II), (III) and (IV).

In the crystal structure, the molecules are linked by N—H···N, N—H···O and C—H···S hydrogen bonds (Table 1) forming a three-dimemsional network (Fig. 2).

### Experimental

An aqueous solution (10 ml) of ammonium thiocyanate (0.152 g, 2 mmol) was added into a beaker containing oxalate acid (0.126 g, 1 mmol) and ethylenediamine (2 mmol) in distilled water (40 ml). After one week of evaporation at room temperature, colourless crystals of the title compound were obtained (yield 92%; m.p. 457.1-458.3 K).

### Refinement

After their location in a difference map, the methylene and ammonium H-atoms were positioned geometrically [N–H = 0.89 Å and C–H = 0.97 Å] and allowed to ride on the parent atoms, with U_iso_(H) = 1.2U_eq_(C,N). A rotating group model was used for the ammonium group.

### Crystal data

**Table:**

C_2_H_10_N_2_^2+^·0.5C_2_O_4_^2^^−^·NCS^−^	*Z* = 2
*M**_r_* = 164.21	*F*(000) = 174
Triclinic, *P*1	*D*_x_ = 1.452 Mg m^−^^3^
Hall symbol: -P 1	Mo *K*α radiation, λ = 0.71073 Å
*a* = 6.4044 (19) Å	Cell parameters from 3525 reflections
*b* = 6.6199 (19) Å	θ = 2.2–28.3°
*c* = 9.377 (3) Å	µ = 0.38 mm^−^^1^
α = 80.799 (5)°	*T* = 298 K
β = 81.179 (5)°	Block, colourless
γ = 74.452 (5)°	0.43 × 0.41 × 0.35 mm
*V* = 375.5 (2) Å^3^	

### Data collection

**Table:**

Bruker SMART APEX CCD area-detector diffractometer	1867 independent reflections
Radiation source: fine-focus sealed tube	1718 reflections with *I* > 2σ(*I*)
graphite	*R*_int_ = 0.017
Detector resolution: 83.66 pixels mm^-1^	θ_max_ = 28.3°, θ_min_ = 2.2°
ω scan	*h* = −8→8
Absorption correction: multi-scan (*SADABS*; Sheldrick, 1996)	*k* = −8→8
*T*_min_ = 0.854, *T*_max_ = 0.879	*l* = −12→12
5091 measured reflections	

### Refinement

**Table:**

Refinement on *F*^2^	Secondary atom site location: difference Fourier map
Least-squares matrix: full	Hydrogen site location: inferred from neighbouring sites
*R*[*F*^2^ > 2σ(*F*^2^)] = 0.034	H-atom parameters constrained
*wR*(*F*^2^) = 0.095	*w* = 1/[σ^2^(*F*_o_^2^) + (0.0453*P*)^2^ + 0.1426*P*] where *P* = (*F*_o_^2^ + 2*F*_c_^2^)/3
*S* = 1.06	(Δ/σ)_max_ = 0.001
1867 reflections	Δρ_max_ = 0.51 e Å^−^^3^
94 parameters	Δρ_min_ = −0.52 e Å^−^^3^
0 restraints	Extinction correction: *SHELXL97*, Fc^*^=kFc[1+0.001xFc^2^λ^3^/sin(2θ)]^-1/4^
Primary atom site location: structure-invariant direct methods	Extinction coefficient: 0.41 (2)

### Special details

**Table:**

Geometry. All esds (except the esd in the dihedral angle between two l.s. planes) are estimated using the full covariance matrix. The cell esds are taken into account individually in the estimation of esds in distances, angles and torsion angles; correlations between esds in cell parameters are only used when they are defined by crystal symmetry. An approximate (isotropic) treatment of cell esds is used for estimating esds involving l.s. planes.
Refinement. Refinement of *F*^2^ against ALL reflections. The weighted *R*-factor wR and goodness of fit *S* are based on *F*^2^, conventional *R*-factors *R* are based on *F*, with *F* set to zero for negative *F*^2^. The threshold expression of *F*^2^ > σ(*F*^2^) is used only for calculating *R*-factors(gt) etc. and is not relevant to the choice of reflections for refinement. *R*-factors based on *F*^2^ are statistically about twice as large as those based on *F*, and *R*- factors based on ALL data will be even larger.

### Fractional atomic coordinates and isotropic or equivalent isotropic displacement parameters (Å^2^)

**Table:**

	*x*	*y*	*z*	*U*_iso_*/*U*_eq_	
O1	0.23176 (14)	0.09074 (15)	0.45456 (11)	0.0325 (2)	
O2	0.41295 (15)	0.23958 (14)	0.57426 (10)	0.0309 (2)	
C1	0.39758 (18)	0.09557 (17)	0.50846 (12)	0.0232 (2)	
S1	0.76420 (7)	0.30131 (6)	0.20864 (5)	0.04886 (18)	
N1	0.7143 (2)	0.7005 (2)	0.04926 (17)	0.0521 (4)	
C2	0.7374 (2)	0.5334 (2)	0.11335 (15)	0.0362 (3)	
N2	0.2850 (2)	0.94792 (18)	0.17891 (12)	0.0346 (3)	
H2A	0.2477	1.0605	0.1135	0.041*	
H2B	0.4190	0.8736	0.1513	0.041*	
H2C	0.2819	0.9898	0.2650	0.041*	
N3	0.18949 (17)	0.64628 (17)	0.44290 (12)	0.0287 (2)	
H3A	0.2241	0.5223	0.4981	0.034*	
H3B	0.0574	0.7188	0.4760	0.034*	
H3C	0.2868	0.7191	0.4460	0.034*	
C3	0.1898 (2)	0.6105 (2)	0.29020 (15)	0.0316 (3)	
H3D	0.0877	0.5264	0.2881	0.038*	
H3E	0.3340	0.5311	0.2548	0.038*	
C4	0.1284 (2)	0.8139 (2)	0.19058 (15)	0.0356 (3)	
H4A	0.1213	0.7813	0.0945	0.043*	
H4B	−0.0157	0.8932	0.2262	0.043*	

### Atomic displacement parameters (Å^2^)

**Table:**

	*U*^11^	*U*^22^	*U*^33^	*U*^12^	*U*^13^	*U*^23^
O1	0.0227 (4)	0.0324 (5)	0.0425 (5)	−0.0015 (3)	−0.0087 (4)	−0.0100 (4)
O2	0.0302 (5)	0.0241 (4)	0.0388 (5)	−0.0025 (3)	−0.0072 (4)	−0.0091 (4)
C1	0.0220 (5)	0.0207 (5)	0.0251 (5)	−0.0035 (4)	−0.0018 (4)	−0.0013 (4)
S1	0.0510 (3)	0.0346 (2)	0.0483 (3)	0.00328 (17)	0.00029 (18)	0.00231 (16)
N1	0.0485 (8)	0.0464 (8)	0.0511 (8)	−0.0062 (6)	−0.0010 (6)	0.0098 (6)
C2	0.0303 (6)	0.0399 (7)	0.0327 (7)	−0.0011 (5)	−0.0009 (5)	−0.0037 (5)
N2	0.0409 (6)	0.0288 (5)	0.0300 (5)	−0.0051 (5)	−0.0044 (4)	0.0023 (4)
N3	0.0267 (5)	0.0258 (5)	0.0341 (6)	−0.0077 (4)	−0.0060 (4)	−0.0002 (4)
C3	0.0323 (6)	0.0261 (6)	0.0353 (7)	−0.0050 (5)	−0.0018 (5)	−0.0067 (5)
C4	0.0396 (7)	0.0336 (7)	0.0332 (7)	−0.0053 (5)	−0.0116 (5)	−0.0031 (5)

### Geometric parameters (Å, °)

**Table:**

O1—C1	1.2539 (15)	N3—C3	1.4879 (18)
O2—C1	1.2474 (15)	N3—H3A	0.89
C1—C1^i^	1.568 (2)	N3—H3B	0.89
S1—C2	1.6295 (16)	N3—H3C	0.89
N1—C2	1.155 (2)	C3—C4	1.5054 (19)
N2—C4	1.4890 (19)	C3—H3D	0.97
N2—H2A	0.89	C3—H3E	0.97
N2—H2B	0.89	C4—H4A	0.97
N2—H2C	0.89	C4—H4B	0.97
			
O2—C1—O1	125.46 (11)	H3A—N3—H3C	109.5
O2—C1—C1^i^	117.42 (13)	H3B—N3—H3C	109.5
O1—C1—C1^i^	117.12 (13)	N3—C3—C4	112.50 (11)
N1—C2—S1	177.95 (14)	N3—C3—H3D	109.1
C4—N2—H2A	109.5	C4—C3—H3D	109.1
C4—N2—H2B	109.5	N3—C3—H3E	109.1
H2A—N2—H2B	109.5	C4—C3—H3E	109.1
C4—N2—H2C	109.5	H3D—C3—H3E	107.8
H2A—N2—H2C	109.5	N2—C4—C3	113.05 (11)
H2B—N2—H2C	109.5	N2—C4—H4A	109.0
C3—N3—H3A	109.5	C3—C4—H4A	109.0
C3—N3—H3B	109.5	N2—C4—H4B	109.0
H3A—N3—H3B	109.5	C3—C4—H4B	109.0
C3—N3—H3C	109.5	H4A—C4—H4B	107.8
			
N3—C3—C4—N2	62.64 (15)		

Symmetry codes: (i) −*x*+1, −*y*, −*z*+1.

### Hydrogen-bond geometry (Å, °)

**Table:**

*D*—H···*A*	*D*—H	H···*A*	*D*···*A*	*D*—H···*A*
N2—H2B···N1	0.89	2.11	2.986 (2)	169
N3—H3A···O2	0.89	2.02	2.8685 (17)	158
C3—H3E···S1	0.97	2.77	3.7294 (18)	169
N2—H2A···N1^ii^	0.89	2.05	2.899 (2)	159
N2—H2C···O1^iii^	0.89	1.95	2.8337 (18)	172
N3—H3B···O1^iv^	0.89	2.02	2.9078 (17)	174
N3—H3C···O1^iii^	0.89	2.40	3.0465 (18)	129
N3—H3C···O2^v^	0.89	1.99	2.8189 (18)	154
C4—H4B···S1^vi^	0.97	2.68	3.4495 (18)	136

Symmetry codes:  (ii) −*x*+1, −*y*+2, −*z*; (iii) *x*, *y*+1, *z*; (iv) −*x*, −*y*+1, −*z*+1; (v) −*x*+1, −*y*+1, −*z*+1; (vi) *x*−1, *y*+1, *z*.
